# Most prevalent unmet supportive care needs and quality of life of breast cancer patients in a tertiary hospital in Malaysia

**DOI:** 10.1186/s12955-016-0428-4

**Published:** 2016-02-22

**Authors:** Zobaida Edib, Verasingam Kumarasamy, Norlia binti Abdullah, A. M. Rizal, Sami Abdo Radman Al-Dubai

**Affiliations:** Department of Community Medicine, International Medical University, Bukit Jalil, 57000 Kuala Lumpur Malaysia; Department of Surgery, Universiti Kebangsaan Malaysia Medical Centre, Cheras, 56000 Kuala Lumpur Malaysia; Department of Community Health, Faculty of Medicine, Universiti Kebangsaan Malaysia Medical Centre, Cheras, 56000 Kuala Lumpur Malaysia; Faculty of Medicine, SEGi University, Kota Damansara, 47810 Petaling Jaya, Selangor Malaysia

**Keywords:** Supportive care, Unmet needs, Quality of life, Breast cancer

## Abstract

**Background:**

Addressing breast cancer patients’ unmet supportive care needs in the early stage of their survivorship have become a prime concern because of its significant association with poor quality of life (QOL), which in turn increases healthcare utilization and costs. There is no study about unmet supportive care needs of breast cancer patients in Malaysia. This study aims to assess the most prevalent unmet supportive care needs of Malaysian breast cancer patients and the association between QOL and patients’ characteristics, and their unmet supportive care needs.

**Methods:**

A cross-sectional study was conducted at the Surgery and Oncology Clinic between May 2014 and June 2014 in a tertiary hospital in Malaysia. A total of 117 patients out of 133 breast cancer patients recruited by universal sampling were interviewed using a structured questionnaire consisted of three parts: participants’ socio-demographic and disease characteristics, Supportive Care Needs Survey-Short Form Questionnaire (SCNS-SF34) and European Organization for Research and Treatment of Cancer Quality of Life Questionnaire C30 (EORTC QLQ-C30).

**Results:**

The highest unmet supportive care needs were observed in the psychological domain (Mean 53.31; SD ± 21.79), followed by physical domain (Mean 38.16; SD ± 27.15). Most prevalent unmet supportive care needs were uncertainty about the future (78.6 %), fears about the cancer spreading (76.1 %), feelings of sadness (69.2 %), feelings about death and dying (68.4 %), concerns about those close to the patient (65.0 %) and feeling down or depressed (65.0 %). Multivariate linear analysis showed that early breast cancer survivors diagnosed at an advanced stage and with greater physical and psychological needs were significantly (*p* < 0.05) associated with poorer QOL.

**Conclusion:**

Most prevalent unmet needs among Malaysian breast cancer patients were found in the psychological domain. Early breast cancer survivors with late stage diagnosis who had more unmet needs in psychological and physical domains were more likely to have a poor QOL.

## Background

Worldwide the most frequently diagnosed cancer among women is the cancer of breast [1]. About half of the breast cancer cases and 60 % of the deaths are estimated to occur in economically developing countries [2]. The breast cancer incidence rates in Malaysia has increased during last three decades at an alarming rate and become an inevitable threat to women. It is estimated that one in 19 women in Malaysia are at lifetime risk, compared to one in 8 in Europe and the United States [2]. Increased awareness, early detection, combination treatment of chemotherapy, irradiation, hormone therapy and advancement of target therapies, as well as better characterization of prognostic factors have remarkably improved the survival rate of women with breast cancer worldwide [3, 4]. The five-year survival rate in the west is 70 to 90 %, in developing countries 57 % and globally 61 % [5, 6]. In Malaysia, the five-year survival rate for breast cancer is 49 % with median interval of 68.1 months and is continuing to escalate every year [7].

Cancer survivorship is an unremitting struggle as the consequence of complex treatment process and it’s multitude of residual and late emerging side effects that have significant impact on physical, psychological, sexual, social and sometimes financial disturbances throughout the post-treatment phase [8–11]. This is considered to be a major aspect that gives rise to multiple unmet needs for breast cancer survivors [12]. Supportive care helps a cancer patient cope with the disease throughout the process of diagnosis, treatment and post-treatment phases [13]. Supportive care is defined as rendering essential services that satisfy cancer patients’ physical, psychological, social, informational and spiritual needs over the entire illness trajectory [14, 15]. Although it is acknowledged as an essential service, 1–93 % of cancer patients’ supportive needs have been consistently unmet [15, 16]. There is growing evidence that the needs perceived by breast cancer patients and the support being provided by the healthcare professionals are diverse [8, 17, 18]. Hence, it is found that the breast cancer patients mostly suffer from physical, emotional, social, financial and psychological disturbances to a greater extent and unmet needs were highest in the post-treatment phase compared to the other phases of cancer continuum [10, 15, 19, 20]. Supportive care is an essential buffering component of cancer patients that helps to regain emotional stability, social adjustment, cognitive function, body image, future perspective and physical strength [21–24].

Understanding the full impact of unmet needs of the breast cancer patients on their QOL is crucial and clinically of prime importance throughout their continuum of survivorship to offer timely effective interventions. Many studies have found that most unmet needs were in the early stage of cancer survivorship that had detrimental effects on QOL of cancer patients [25–27]. However, several studies suggesting that greater supportive care was associated with long survival and better QOL [27–29]. Thus improving the QOL of breast cancer patients requires addressing the unmet supportive care needs of the breast cancer patients [30]. Assessing needs also offer a direct measure of the patients’ support preference and service gaps [31]. Studying the perception of the breast cancer patients’ unmet supportive care needs clarifies where actions and resource allocation are necessary in healthcare setting to help the patients to overcome their difficulties.

Effective high quality management in healthcare setting is accounted as more than just delivery of anti-cancer therapy [22, 32]. However, it focuses on identification of unsatisfied needs of cancer patients that provides the opportunity to enhance the QOL, which in turn also reduces health care utilization and costs [33–37]. Evaluating these unmet supportive care needs of the breast cancer patients enables healthcare providers to identify those which lack the level of service or support they perceive essential to achieve the optimal quality of life (QOL). In this context, addressing breast cancer patients’ unmet needs in the early stage of their survivorship provides rationale to enhance their QOL and guidance for new strategies in healthcare setting that could potentially reduce the burden of this disease and treatment in the long run and thereby improve their QOL. Existing knowledge on unmet supportive care needs of the breast cancer patients and QOL is predominantly from the western countries. However, there is no evidence of addressing unmet supportive care needs of breast cancer patients and how it is associated with QOL in Malaysia. This study aims to assess the most prevalent unmet supportive care needs of breast cancer patients and the association between QOL and these patients’ characteristics, and their unmet supportive care needs in a tertiary hospital in Malaysia.

## Methods

### Study design and sample

A cross-sectional study was conducted at the Surgery and Oncology Clinic on Clinic Day in University Kebangsaan Malaysia Medical Centre (UKMMC) between May 2014 and June 2014 among breast cancer patients. Inclusion criteria for recruiting the patients were: female primary and recurrent breast cancer patients of all ages and with any stages, who had survived at least one year after being diagnosed by a registered physician, must be Malaysian and who can speak either English or Malay. Excluded from this study were those who had secondary breast cancer and were terminally ill and those who were not able to speak.

### Instruments

A structured questionnaire was used in this study which consisted of the following parts: participants’ socio-demographic and disease characteristics, Supportive Care Needs Survey -Short Form (SCNS-SF 34) and European Organization for Research and Treatment of Cancer Quality of Life Questionnaire C30 (EORTC QLQ-C30). SCNS-SF34 is a standardised instrument for measuring cancer patients’ perceived needs across a range of domains. A total number of 34-items are divided into five domains: physical/daily living (5 items), psychological (10 items), sexuality (3 items), patient care and support (5 items) and health system and information needs (10 items). Internal consistency was high with Cronbach’s alpha coefficients for the five domains ranging from 0.87 to 0.96 [38]. A five-point rating scale (1 = no need/not applicable, 2 = no need/satisfied, 3 = low need, 4 = moderate need and 5 = high need) is used to rate the levels of need over the past month. Standardised likert summated score was used to score SCNS-SF34 according to Supportive Care Needs Survey scoring manual. The score has possible values ranging from 0 to 100, with a higher score indicating more unmet needs [39]. EORTC QLQ-C30 is a standardized questionnaire which was constructed by the EORTC Quality of Life Study Group to measure the quality of life of cancer patients. The linear transformation of the raw score of the global QOL was done according to EORTC scoring manual to standardize the raw score so that it ranges from 0 to100 [40]. The correlation coefficient for global QOL was 0.85 [41].

The Malay version of the questionnaire was prepared for the participants who cannot speak English. The questionnaire was translated into Malay language by an independent language expert who was not associated with this study. This Malay version of the questionnaire was then back-translated into English by another independent language expert not associated with this study. This second English version of the questionnaire was then re-translated into Malay by another independent language expert not associated with this study. Both the two sets of English and Malay version were subsequently compared with the original version by the Breast and Reconstructive Surgeons for the conceptual equivalence of the items. Then the final questionnaire was verified by the Surgery Department of UKMMC and the Community Medicine Department of International Medical University. Minor modifications were made wherever applicable to the translated questionnaires to remove discrepancies. Pre-testing was done on a sample of eight breast cancer patients at the Surgery Clinic of UKMMC to assess the acceptance and time management.

### Ethical consideration

The study was approved by Medical Ethics Committee of International Medical University (Project Number: M.ScPHI01/2014(01) and Research Ethics Committee of Universiti Kebangsaan Malaysia Medical Centre (Approval Number: 1.5.3.5/244/FF-2014-255).

### Procedure

A convenience sample of 133 breast cancer patients defined by the sampling criteria were recruited from the Surgery and Oncology out-patient clinic of UKMMC. The study was conducted on a voluntary basis where the selected participants voluntarily agreed to take part in this study. Out of total 133 breast cancer patients, 117 patients voluntarily agreed to take part in the study and gave their written informed consent. Participants were well informed about the purpose of the study and reassured that all the personal information will be kept confidential, and they could withdraw their consent anytime without giving any reason. In addition to that, briefing on the questionnaire in both English and Malay was conducted to ensure the accuracy of collecting information. The participants were allowed to enquire any questions related to the questionnaire. Data were obtained through interviewer-administered questionnaire to ensure the quality of collected information and reduce the refusal rate. Two trained interviewers were assigned for the briefing of the study, securing written informed consent and for conducting the interviews under the direct supervision of the researcher. However, the interviewers and the researcher had no relation with the participants.

### Data analysis

Data were tabulated and analysed by using the Statistical Package for the Social Sciences version 20.0 (SPSS Inc.; Chicago, IL, USA). Descriptive analysis was used for demographic and disease characteristics as well as supportive care needs items. Mean, median scores and standard deviation (SD) were calculated for supportive care needs’ domains. Univariate analyses of the relationship between the global QOL and domains of supportive care needs, and participants’ socio-demographic and disease characteristics were examined by the means of independent *t*-test, one way analysis of variance (ANOVA) and Pearson correlation coefficient, as appropriate. All variables with *p* value < 0.25 in univariate analyses were chosen for multiple linear regression to determine the variables that were independently associated with global QOL. All tests of significance were two-sided and with *p* value < 0.05 was considered as statistically significant.

## Results

### Socio-demographic and disease characteristics of the participants

Total 117 patients were agreed to participate in this study out of 133 breast cancer patients defined by the inclusion and exclusion criteria (response rate = 88.0 %). A summary of the participants’ socio-demographic and disease characteristics are provided in the Table 1. More than half of the participants aged 50 years and above (61.6 %). The majority were Malay (58.1 %), followed by Chinese (29.9 %) and Indian (12.0 %). About 13 % of the participants reported that they had no formal education and 16.3 % had only primary education, while almost one-third (31.6 %) had a tertiary level of education. The majority of the participants were married (77.8 %). Of the total participants, half of them were housewife (53.8 %) followed by employed (31.6 %) and retired (14.5 %). Among the participants 41.0 % had a household income between RM2000 and RM4000.

**Table 1:** Socio-demographic and disease characteristics of participants (*n* = 117)

Socio-demographic and disease characteristics	Total sample (117)	
	N	%
Age		
< 40 years	16	13.7
40–49 years	29	24.8
> 50 years	72	61.6
Ethnicity		
Malay	68	58.1
Chinese	35	29.9
Indian	14	12.0
Educational level		
No formal education	15	12.8
Primary	19	16.3
Secondary	46	39.3
Tertiary	37	31.6
Marital status		
Unmarried	4	3.4
Married	91	77.8
Divorced/widowed	22	18.8
Employment status		
Housewife	63	53.8
Employed	37	31.6
Retired	17	14.5
Household income (RM)		
Less than 2000	41	35.1
2000 ─ 4000	48	41.0
More than 4000	28	23.9
Time since diagnosis		
< 2 years	50	42.7
2–5 years	50	42.7
> 5 years	17	14.6
Stage at diagnosis		
Stage 0	8	6.8
Stage I	24	20.5
Stage II	43	36.8
Stage III	28	23.9
Stage IV	14	12.0
Type of surgery		
Breast conserving surgery	37	31.6
Mastectomy	80	68.4
Radiotherapy		
Yes	94	80.3
No	23	19.7
Chemotherapy		
Yes	84	71.8
No	33	28.2
Hormone therapy^a^		
Yes	92	79.3
No	24	20.7
Immune therapy^a^		
Yes	26	22.6
No	89	77.4

^a^Number of participants less than 117 (total respondents) due to non-response

About 43.0 % of the participants had been diagnosed with breast cancer less than 2 years ago. More than one-third of the participants (36.8 %) were diagnosed at stage II, followed by 23.9 % at stage III. While 20.5 % was diagnosed with stage I and others in stage IV. Regarding the treatment modalities, the majority of the participants (68.4 %) underwent mastectomy, 80.3 % received radiotherapy, 71.8 % had chemotherapy and 79.3 % had hormone therapy.

### Most common unmet supportive care needs

The most commonly reported unmet supportive care needs were found all in the psychological domain, followed by physical domain. Table 2 represents the percentages of some unmet supportive care needs of individual items of SCNS-SF 34 among the participants. Most prevalent unmet supportive care needs in the psychological domain were uncertainty about the future (78.6 %), followed by fears about the cancer spreading (76.1 %), feelings of sadness (69.2 %), feelings about death and dying (68.4 %), concerns about the worries of those close to the patient (65.0 %), worries that the results of treatment are beyond control (65.0 %) and feeling down or depressed (65.0 %). Most prevalent unmet supportive care needs in the physical domain were feeling unwell a lot of the time (58.1 %), followed by lack of energy/tiredness (57.3 %) and pain (55.6 %). In the sexuality domain the most prevalent unmet supportive care need was changes in sexual relationships (35.0 %). In the patient care domain and health system information domain, the percentage of some unmet supportive care needs was very low except the choices about specialists the patients see (45.3 %).

**Table 2:** Some unmet supportive care needs of individual item of SCNS-SF 34 among the study sample

Some unmet supportive care needs	N (%)
Physical	
Pain	65 (55.6)
Lack of energy/tiredness	67 (57.3)
Feeling unwell a lot of the time	68 (58.1)
Work around the home	45 (38.5)
Not being able to do the things you used to do	43 (36.8)
Psychological	
Anxiety	65 (55.6)
Feeling down or depressed	76 (65.0)
Feelings of sadness	81 (69.2)
Fears about the cancer spreading	89 (76.1)
Worry that the results of treatment are beyond your control	76 (65.0)
Uncertainty about the future	92 (78.6)
Learning to feel in control of your situation	55 (47.0)
Keeping a positive outlook	60 (51.3)
Feelings about death and dying	80 (68.4)
Concerns about the worries of those close to you	76 (65.0)
Sexuality	
Changes in sexual feelings	40 (34.2)
Changes in your sexual relationships	41 (35.0)
Being given information about sexual relationships	25 (21.4)
Patient Care	
More choice about which cancer specialists you see	53 (45.3)
More choice about which hospital you attend	42 (35.9)
Reassurance by medical staff that the way you feel is normal	38 (32.5)
Hospital staff attending promptly to your physical needs	35 (29.9)
Hospital staff acknowledging, and showing sensitivity to, your feelings and emotional needs	45 (38.5)
Health System Information	
Being given written information about the important aspects of your care	14 (12.0)
Being given information (written, diagrams, drawings) about aspects of managing your illness and side-effects at home	32 (27.4)
Being given explanations of those tests for which you would like explanations	35 (29.9)
Being adequately informed about the benefits and side-effects of treatments before you choose to have them	27 (23.1)
Being informed about your test results as soon as feasible	14 (12.0)
Being informed about cancer which is under control or diminishing (that is, remission)	24 (20.5)
Being informed about things you can do to help yourself to get well	21 (17.9)
Having access to professional counselling (e.g. psychologist, social worker, counsellor, nurse specialist) if you, family or friends need it	17 (14.5)
Being treated like a person not just another case	25 (21.4)
Being treated in a hospital or clinic that is as physically pleasant as possible	20 (17.1)
Having one member of hospital staff with whom you can talk to about all aspects of your condition, treatment and follow-up	16 (13.7)

Table 3 summarizes the mean and median score of the supportive care needs scale of SCNS-SF 34 among the participants. Among all the supportive care needs domain of SCNS-SF 34, psychological needs were observed to have the highest mean (53.31 ± 21.79), followed by physical needs (38.16 ± 27.15). The lowest mean score domain was observed in sexuality (27.78 ± 21.91). The mean score for the patient care needs was 37.65 (±16.45) and for health information needs was 31.53 (±12.17).

**Table 3:** Mean and median score of supportive care needs scale of SCNS-SF 34 among the study population

Variables	Mean (+SD)	Median (Range)
Physical needs	38.16 (27.15)	40 (0–100)
Psychological needs	53.31 (21.79)	52.50 (10–92.50)
Sexuality needs	27.78 (21.91)	25 (0–91.67)
Patient care needs	37.65 (16.45)	30 (25–85)
Health information needs	31.53 (12.17)	25 (20.45–77.27)

### Association between global QOL, participants’ characteristics and supportive care needs

Participants’ socio-demographic and disease characteristics as well as their supportive care needs were examined for their association with global QOL (Table 4). Multiple linear regression analyses, using those variables with *p* values < 0.25 in univariate analyses as candidate variables, revealed that time since diagnosis (β = 0.177; *p* value = 0.003), stage at diagnosis of breast cancer (β = -0.215; *p* value = 0.008), physical (β = -0.346; *p* value = 0.001) and psychological unmet needs (β = -0.218; *p* value = 0.004) were independently associated with QOL among the breast cancer patients. Early breast cancer survivors with advanced stage diagnosis who had greater physical and psychological needs were significantly (*p* < 0.05) associated with poor QOL. The adjuste *R*^2^ for this model was 0.780 that means 78.0 % variability of the outcome is explained by this model. The regression model was highly significant, *p* <0.001.

**Table 4:** Association between global quality of life, participants’ characteristics and supportive care needs

Variables	Univariate analysis		Multivariate analysis^a^			
	Mean (SD)	*p* value	B	SE	β	*p* value
Socio-demographic and disease characteristics						
Age			−0.282	1.647	−0.011	0.864
< 40 years	79.69 (12.53)					
40–49 years	71.26 (18.44)	0.004				
> 50 years	63.77 (19.08)					
Ethnicity			−0.038	1.452	−0.002	0.979
Malay	61.15 (18.64)					
Chinese	76.19 (14.23)	0.001				
Indian	77.38 (17.86)					
Educational Level			2.785	1.912	0.146	0.148
No formal education	44.44 (12.06)					
Primary	58.33 (20.41)	0.001				
Secondary	68.12 (15.34)					
Tertiary	81.76 (10.90)					
Marital Status			0.941	2.592	0.022	0.717
Unmarried	72.92 (15.77)					
Married	69.96 (18.54)	0.023				
Divorced/widowed	57.95 (18.62)					
Employment Status			−2.736	2.256	−0.104	0.228
Housewife	58.46 (17.74)					
Employed	81.30 (11.01)	0.001				
Retired	73.04 (17.56)					
Household Income (RM)			3.231	2.176	0.130	0.141
Less than 2000	50.61 (13.61)					
RM2000- 4000	74.48 (15.41)	0.001				
More than 4000	81.55 (11.19)					
Time since Diagnosis			4.746	1.573	0.177	0.003
< 2 years	62.83 (19.21)					
2–5 years	67.00 (18.43)	0.001				
> 5 years	84.80 (6.06)					
Stage at Diagnosis			−3.776	1.385	−0.215	0.008
Stage 0	79.16 (7.71)					
Stage 1	77.08 (12.83)					
Stage 2	70.73 (17.19)	0.001				
Stage 3	65.18 (19.51)					
Stage 4	41.67 (10.34)					
Type of Surgery			−1.668	2.947	−0.041	0.573
Breast conserving surgery	74.32 (12.79)	0.011				
Mastectomy	64.79 (20.54)					
Radiotherapy			3.499	4.999	0.074	0.486
Yes	64.45 (19.40)	0.001				
No	81.52 (7.08)					
Chemotherapy			−5.305	4.173	−0.127	0.207
Yes	62.79 (19.59)	0.001				
No	80.55 (8.24)					
Hormone therapy			3.502	4.020	0.075	0.386
Yes	64.67 (19.43)	0.001				
No	80.56 (9.41)					
Immune therapy			−3.396	2.963	−0.075	0.255
Yes	75.96 (17.69)	0.013				
No	65.54 (18.81)					
Unmet Supportive Care Needs	Correlation coefficient					
Physical Needs	−0.76	0.001	−0.241	0.070	−0.346	0.001
Psychological Needs	−0.80	0.001	−0.191	0.094	−0.218	0.004
Sexuality Needs	−0.06	0.553	NS	NS	NS	NS
Patient’s Care Needs	−0.44	0.001	−0.028	0.103	−0.024	0.787
Health Information Needs	−0.16	0.092	0.009	0.122	0.006	0.941

^a^Multiple linear regression model included 117 participants with complete covariate information

B: Regression coefficient, SE: Standard error of regression coefficient, β: Standardized regression coefficient, NS: Not significant and not being entered into multiple linear regression (*p* value > 0.25), *p* value < 0.05 is considered as statistically significant

In summary, the most common unmet supportive care needs were found to be in the psychological domain. Early breast cancer survivors, diagnosed at an advanced stage who had greater physical and psychological needs were likely to have a poorer quality of life.

## Discussion

This is the first study addressing the supportive care needs of breast cancer patients in Malaysia. It reveals a multitude of unmet supportive care needs of breast cancer patients in all the domains with highest prevalence in the psychological domain, followed by physical, patient care needs, health information and lowest in sexuality domain. The most prevalent unmet psychological needs of the breast cancer patients reported in this study were uncertainty about the future, fears about the cancer spreading, feelings of sadness, feelings about death and dying, concerns about those close to the patient, worry that the results of treatment are beyond control and feeling down or depressed. In western countries unmet needs were highest in psychological domain which is consistent with the findings of this study [36, 42, 43]. Whereas in Asian developing countries unmet needs were mostly related to health system information [17, 44–47]. As a matter of fact, supportive care needs are the product of perspective of culture and interaction of psychology based on cultural context [30]. With the westernization, the perception of cancer and unmet needs of the cancer patients has been changing over few decades in Malaysia [48]. It should also be noted that most of the breast cancer patients in this study had completed their initial course of treatment less than 5 years ago and it is possible that the psychological supportive care needs had not dissipated by the time they participated in the study. It has found that psychological distress of breast cancer patients were higher among those with survival duration of less than 5 years than those with long-term survival more than 5 years [29]. Nevertheless, in Malaysian healthcare settings, providing services of psychosocial components of care for cancer patients are not yet well established [48]. Incorporation of psychosocial components of care in the routine cancer care delivery in healthcare settings is crucial and can be challenging as there is no existing training, guidelines and strategies for the healthcare providers in Malaysia. Studies suggest that addressing psychological needs can have a great role in helping women with breast cancer in the long-term adjustment process and improving their QOL [28, 49, 50].

Particularly amongst various types of physical needs, breast cancer patients had stronger needs in their physical strength and dealing with pain. These could be the residual side effects or late onset symptoms of breast cancer treatment which should be monitored timely to characterize the level of unmet needs over time. There is a growing volume of literatures which support the proposition that lack of physical strength and pain are the critical physical needs among the breast cancer patients throughout the period of treatment and survivorship [8, 51]. The experience of these persistent symptoms and morbidities as well as associated unmet needs could significantly hamper their QOL and successful transition from early to long-term survivorship [4]. These findings have important clinical implication in providing timely and appropriate physical rehabilitation depending on the needs in healthcare setting after the initial course of breast cancer treatment.

In Malaysian healthcare system, breast cancer patients have a regular access to healthcare professionals during their therapy and in the post treatment period, which develops confidence over the clinical team concerning the treatment [52]. Therefore, the patient care and health system and information needs might seem to be very low except many had a need to have more choices concerning the cancer specialists they see. A possible explanation could be a divergence between their expectations to have more opinions for the assurance that quickly they would cope up and come back to normal life.

Congruent with other studies, it was expected to find sexuality domain as least unmet needs in this study [44, 53]. However, the findings may not reflect the actual status in a conservative society like Malaysia where conservative cultural values consider talking about sexuality is embarrassing [54]. Moreover, this would eventually make patients to perceive that sexuality is a low priority despite having certain sexual problems. Although impairment in sexual functioning has significant negative impact on psychological well-being and QOL [55, 56].

In multivariate analysis, the results showed that early breast cancer survivors diagnosed at an advanced stage with higher levels of physical and psychological unmet needs were more likely to have an overall poor QOL. Breast cancer patients with late stage diagnosis invariably require a lengthy and complicated course of treatment [4, 6, 8]. As seen in other studies, the unanticipated side effects and the struggle through the lengthy and disruptive treatment procedure might give rise to the psychological and physical difficulties to the breast cancer patients [4, 36, 44, 46, 57–59]. It is not unlikely that poor psychological adjustment might enhance physical disability or vice versa [33]. The complicated long-term treatment which led to physical and psychological sufferings makes early breast cancer survivors’ lives miserable. Breast cancer patients certainly need time to get over the burdens of side effects of the long-term treatment [3, 12]. Therefore, continuous and timely psychological support and physical rehabilitation may have contributed to reduce their chronic sufferings, helped them with the adjustment process over and after the course of long-term treatment and could possibly enhance their QOL.

This study had several limitations. The research was conducted to the surgery and oncology outpatients of a single large government academic hospital, which limits the applicability of the findings to the breast cancer patients all over Malaysia. The patients were recruited by universal sampling, which led to sampling bias. Several types of response bias may also be possible while collecting data. Acquiescence bias is one of those where participants tend to answer questions affirmatively when they have doubts. Another is a central tendency bias where participants tend to avoid extreme scores and give response towards the centre of the scale range. Questions on sexuality may not have been answered truthfully due to cultural barriers which may cause information bias.

## Conclusion

The unmet needs among the breast cancer patients were found predominant in the psychological and physical domain. Hence, most prevalent unmet needs were found in the psychological domain. Early breast cancer survivors with late stage diagnosis, who had greater unmet needs in the physical and psychological domains were more likely to have a poor QOL. The research findings provide comprehensive insight into unmet needs of breast cancer patients across a range of domains for well-directed and effective management in clinical-follow up. However, it emphasizes that rendering timely and appropriate psychological and physical rehabilitation programmes in healthcare setting ought to be the highest priority to support them in the long-term adjustment process and ensure a better QOL.
